# Phenotypic and Genotypic Characteristics of *Neisseria meningitidis* Disease-Causing Strains in Argentina, 2010

**DOI:** 10.1371/journal.pone.0058065

**Published:** 2013-03-04

**Authors:** Cecilia Sorhouet-Pereira, Adriana Efron, Paula Gagetti, Diego Faccone, Mabel Regueira, Alejandra Corso, Argentinean SIREVA II Working Group, Jean-Marc Gabastou, Ana Belén Ibarz-Pavón

**Affiliations:** 1 Clinical Bacteriology Service, Department of Bacteriology, National Institute for Infectious Diseases (ANLIS-INEI), ‘Dr Carlos G. Malbrán’, Ministry of Health, Buenos Aires, Argentina; 2 Antimicrobial Resistance Service, Department of Bacteriology, National Institute for Infectious Diseases (ANLIS-INEI), ‘Dr Carlos G. Malbrán’, Ministry of Health, Buenos Aires, Argentina; 3 Pan American Health Organization, Washington, DC, United States of America; University of Cambridge, United Kingdom

## Abstract

Phenotypic and genotypic characterization of 133 isolates of *Neisseria meningitidis* obtained from meningococcal disease cases in Argentina during 2010 were performed by the National Reference Laboratory as part of a project coordinated by the PAHO within the SIREVA II network. Serogroup, serotype, serosubtype and MLST characterization were performed. Minimum Inhibitory Concentration to penicillin, ampicillin, ceftriaxone, rifampin, chloramphenicol, tetracycline and ciprofloxacin were determined and interpreted according to CLSI guidelines. Almost 49% of isolates were W135, and two serotype:serosubtype combinations, W135∶2a:P1.5,2:ST-11 and W135∶2a:P1.2:ST-11 accounted for 78% of all W135 isolates. Serogroup B accounted for 42.1% of isolates, and was both phenotypically and genotypically diverse. Serogroup C isolates represented 5.3% of the dataset, and one isolate belonging to the ST-198 complex was non-groupable. Isolates belonged mainly to the ST-11 complex (48%) and to a lesser extent to the ST-865 (18%), ST-32 (9,8%) and the ST-35 complexes (9%). Intermediate resistance to penicillin and ampicillin was detected in 35.4% and 33.1% of isolates respectively. Two W135∶2a:P1.5,2:ST-11:ST-11 isolates presented resistance to ciprofloxacin associated with a mutation in the QRDR of gyrA gene Thr91-Ile. These data show serogroup W135 was the first cause of disease in Argentina in 2010, and was strongly associated with the W135∶2a:P1.5,2:ST-11 epidemic clone. Serogroup B was the second cause of disease and isolates belonging to this serogroup were phenotypically and genotypically diverse. The presence of isolates with intermediate resistance to penicillin and the presence of fluorquinolone-resistant isolates highlight the necessity and importance of maintaining and strengthening National Surveillance Programs.

## Introduction

*Neisseria meningitidis* (NM) is an important cause of bacterial meningitis and sepsis worldwide [1]. The spectrum of human-meningococcal interactions ranges from asymptomatic carriage to fatal infection. Invasive Meningococcal Disease (IMD) mainly comprises two clinically overlapping syndromes: septicæmia and meningitis. The disease can initially be difficult to distinguish from other febrile illnesses, usually develops rapidly and has a high case fatality rate. Survivors often suffer from impairing physical and neurological sequelæ [1], [2].

Laboratory-based surveillance of IMD in Argentina is carried out by a national network comprising 74 participating laboratories distributed across 23 provinces, including Buenos Aires City, and the National Reference Laboratory (NRL) which is part of the twenty participating laboratories in the SIREVA II network (*Sistema de redes de vigilancia de agents bacterianos responsables de neumonías y meningitis*), a laboratory-based bacterial meningitis and pneumonia surveillance network supported and coordinated by the Pan-American Health Organization [3], [4], [5].

The incidence of IMD in Argentina experienced a progressive decline from 2.6/100,000 in 1993 to 0.6/100,000 in 2005 [6], which appears to be sustained to date. Disease-causing serogroups have also changed over the years: whereas serogroup C was the main cause of disease during the 1990s, serogroup W135 became more prevalent from 2001 onwards, and since 2008 this serogroup represents over 50% of meningococcal isolates submitted to the NRL every year [3], [7].

Although phenotypic information on circulating meningococci in Argentina has been published previously [6], data on circulating genotypes is scarce. With the upcoming licensure of antigen-based universal vaccines that are being developed on the basis of genetic data [8], [9], [10], [11], [12], [13], [14], the implementation of a molecular epidemiology-based surveillance is essential to allow public-health authorities to make an evidence-based decision regarding their implementation through the national immunization program.

This paper represents, to the authorś knowledge, the first comprehensive report of disease-causing meningococcal genetic lineages circulating in Argentina over the period of a year. From 1^st^ January to 31^st^ December 2010, a total of 133 meningococcal isolates from an equal number of patients were recovered nationwide by the surveillance network, and sent to the NRL for phenotypic and genotypic characterization, and determination of their susceptibility profile. In the same period of time, 125 cases of disease were reported to the compulsory notification system in the Ministry of Health, and the reported disease incidence rate for 2010 was of 0.03/100.000.

## Materials and Methods

A total of 133 isolates were received by the NRL from 74 laboratories participating in the national surveillance network. Meningococcal isolates are sent in a voluntary basis, and as a rule, when an isolate is recovered from both blood and cerebrospinal fluid (CSF) from a single patient on the same disease episode, only that obtained from CSF is investigated. Long-term storage of isolates is done using a skim-milk and glycerol solution at −70°C.

Stored isolates were retrieved from the freezer, cultured on blood agar plates and incubated in 5% CO_2_ atmosphere at 36°C for 18 h. Multilocus Sequence Typing (MLST) was performed on boiled suspensions as previously described by Maiden *et al*., using the primers listed on *Neisseria* PubMLST website (http://pubmlst.org/neisseria) [15]. Amplified fragments were purified by polyethylene glycol precipitation and sequenced at a dedicated facility. Sequence comparison and analysis were done using the Vector NTI Advance 11 software, and allele, ST and Clonal complex designation were retrieved from the PubMLST website.

A total of 130 out of the 133 isolates were available for serological and antimicrobial susceptibility tests. The serotype and serosubtype determination was performed by whole-cell ELISA against a battery of 21 serotype and serosubtype-specific monoclonal antibodies (MAbs) [16] (provided by the NIBSC Potters Bar, England through the *Instituto Adolfo Lutz*, São Paulo-Brazil).

Minimum Inhibitory Concentration (MIC) to penicillin, ampicillin, ceftriaxone, rifampin, chloramphenicol, tetracycline and ciprofloxacin were performed by agar dilution method and interpreted according to CLSI guidelines [17]. Susceptibility to nalidixic acid was evaluated by disk diffusion method (CLSI M2-A10). β-lactamase assay was performed by Cefinase discs (BBL). The Quinolone Resistance-Determining Region (QRDR) of *gyr*A and *par*C genes were amplified by PCR and sequenced as previously described [18].

## Results

**Figure 1:**
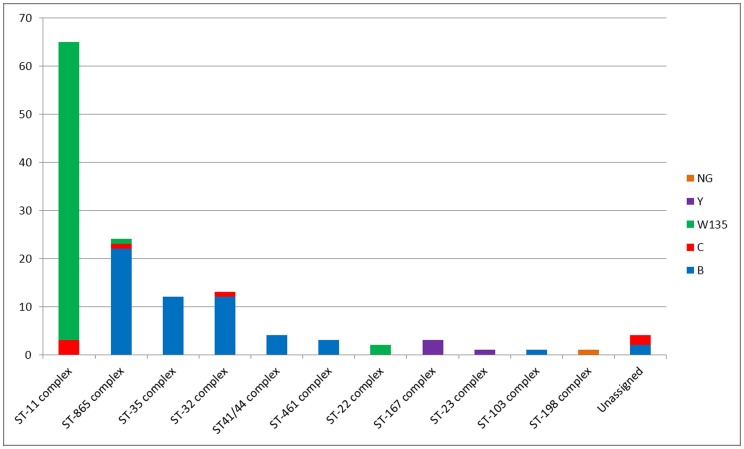
Distribution of serogroups by clonal complex in Argentina, 2010.

Phenotypic and genotypic characteristics of meningococcal isolates are shown on Table 1. Figure 1 shows serogroup distribution among clonal complexes. A total of 65 (48,9%) of the 133 disease-causing isolates for which serogroup was determined belonged to serogroup W135, and 56 (42,1%) belonged to serogroup B. Serogroup C was found in 7 isolates (5.2%), and four (3%) belonged to serogroup Y. One isolate was found to be non-groupable (NG). MLST analysis revealed that 65 isolates (48,8%) belonged to ST-11 complex of which 62 (95,4%) were serogroup W135 and the remaining three were serogroup C. When serological characterization was combined with MLSTW135∶2a:P1.5,2:ST-11 and W135∶2a:P1.2:ST-11 were the most frequent combinations, with 30 and 20 isolates respectively. Two new STs belonging to the ST-11 complex, ST-9434 and ST-9435, were reported to the PubMLST website, and both strains were serogroup W135.

**Table 1:** Characterization of 132 isolates received in de NRL in 2010.

Strain type	N
**ST-11 complex**	
W135∶2a:P1.5,2:ST-11	30
W135∶2a:P1.2:ST-11	20
W135∶2a:NST:ST-11	6
W135∶2a:P1.5:ST-11	2
W135∶2a:P1.5,2:ST-5036	1
W135∶2a:P1.5,2:ST-7579	1
W135∶2a:P1.5,2:ST-9434	1
W135∶2a:P1.5,2:ST-9435	1
C:2a:P1.5:ST-11	2
C:2a:P1.5,2:ST-11	1
**ST-865 complex**	
B:NT:NST:ST-3327	11
B:15:NST:ST-3327	8
B1:NT:NST:ST-3327	1
B:15:P1.22-1,14:ST-3327	1
B:ST-3327^a^	1
C:ST-9442^a^	1
W135:NT:NST:ST-3327	1
**ST-35 complex**	
B:4:P1.22-1,14:ST-35	6
B:NT:P1.22-1,14:ST-35	2
B:4:P1.7:ST-35	1
B:NT:P1.22-1,14:ST-160	1
B:NT:P1.22-1,14:ST-7479	1
B:NT:P1.22-1,14:ST-6402	1
**ST-32 complex**	
B:15:P1.7,16:ST-1880	1
B:NT:P1.7,16:ST-1880	1
B:15:NST:ST-1880	1
B:NT:P1.7,2:ST-1880	1
B:ST-1880^a^	1
B:4:NST:ST-9436	1
B:4:P1.7,16:ST-9438	1
B:15:P1.7,16:ST-9443	2
B:15:P1.7,16:ST-9444	1
B:4:P1.19,15:ST-33	1
B:15:P1.19,15:ST-33	1
C:15:P1.7:ST-32	1
**ST41/44 complex**	
B:1:NST:ST-409	2
B:1:NST:ST-9437	1
B:1:NST:ST-9439	1
**ST-461 complex**	
B:1:NST:NST:ST-1946	2
B:NT:NST:ST-5983	1
**ST-22 complex**	
W135:NT:P1.3:ST-22	1
W135:NT:NST:ST-22	1
**ST-167 complex**	
Y:NT:P1.5:ST-1624	3
**ST-23 complex**	
Y:NT:NST:ST-6519	1
**ST-103 complex**	
B:NT:P1.5,2:ST-103	1
**ST-198 complex**	
NG:15:NST:ST-823	1
**Unassigned**	
B:NT:NST:ST-9433	1
B:NT:NST:ST-9441	1
C:4:NST:ST-9440	2

a unavailable for serological characterization.

Serogroup C isolates belonging to the ST-11 complex presented combinations: C:2a:P1.5:ST-11 (two isolates) and C:2a:P1.5,2:ST-11 (one isolate). A total of 24 (18%) isolates belonged to the ST-865 complex, of which 23 (95.8%) were ST-3327 strains, and the remaining one was the newly reported ST-9442. All but two of the 24 isolates were serogroup B, of which 11 were non-typable (NT) and non-serosubtypable (NST) (B:NT:NST:ST-3327) by ELISA. Thirteen isolates belonged to the ST-32 complex, of which 12 were serogroup B and one, serogroup C. The majority of isolates were ST-1880 and serotype:serosubtype combinations were heterogeneous. Three new STs, ST-9438, ST-9443 and ST-9444 were detected among isolates belonging to ST-32 complex. The ST-35 complex was found in 12 (21,5%) of serogroup B isolates, with most of them presenting the phenotype B:4:P1.22-1,14. Four serogroup B isolates belonged to ST-41/44 complex, of which two, ST-9437 and ST-9439, were identified here for the first time. The other two isolates were B:1:NST:ST-409. Three isolates of the ST-461 complex were found: two had the combination B:1:NST:ST-1946, and the remaining one was B:NT:NST:ST-5983. Only one isolate belonging to the ST-103 complex was found, and was B:NT:P1.5,2:ST-103. Two serogroup C isolates of the newly described ST-9440, which is not yet assigned to any clonal complex, presented the combination C:4:NST:ST-9440. Of the four serogroup Y isolates, three belonged to the ST-167 complex, and were characterized as Y:NT:P1.5:ST-1624 and the last one was Y:NT:NST:ST-6519 and belong to the ST-23 complex. Finally the NG isolate was NG:15:NST:ST-823:ST-198 complex.

Intermediate resistance to penicillin (MIC 0.12–0.25 mg/L) and ampicillin (MIC 0.25–1 mg/L) was detected in 35.4% and 33.1% of the 130 available NM isolates, respectively (Table 2). None of the isolates presented penicillin MIC >0.25 mg/L, and all of them tested negative for the production of β-lactamase. Intermediate resistance to penicillin was detected in: 68.5%, 33.3%, 25% and 10.1% of serogroup B, C, Y and W135 isolates respectively. All strains remained susceptible (MIC_90_ mg/L) to ceftriaxone (0.002), chloramphenicol (1), tetracycline (0.25) and rifampin (0.03) (Table 2). Two W135∶2a:P1.5,2:ST-11:ST-11 complex isolates were resistant to ciprofloxacin with MICs of 0.12 mg/L, showing inhibition zones of 6 and 18 mm against nalidixic acid disk. There was no epidemiological link between these two cases. Both presented the mutation Thr91-Ile in the QRDR of *gyr*A gene (Table 3). These two strains were susceptible to all the others antibiotic tested. Mutations were not found in the QRDR of *par*C gene.

**Table 2:** Antimicrobial susceptibility of 130 *Neisseria meningitidis* isolates received in the NRL in 2010.

Antibiotic	CLSI Break-point	n°(%) of isolates			CIM50 (mg/L)	CIM90 (mg/L)	MIC range (mg/L)
		S	I	R			
Ampicillin	S≤0.12 R≥2	87(66.9)	43(33.1)	0	0.06	0.25	0.015 - 0.5
Penicillin	S≤0.06 R≥0.5	84 (64.6)	46 (35.4)	0	0.06	0.25	0.015 - 0.25
Ceftriaxone	S≤0.12	130(100)	0	0	0.001	0.002	0.0005 - 0.004
Rifampicin	S≤0.5 R≥2	130(100)	0	0	0.008	0.03	≤0.004–0.06
Chloramphenicol	S≤2 R≥8	130(100)	0	0	1	1	0.25–2
Tetracycline	S≤2	130(100)	0	0	0.12	0.25	0.06–0.5
Ciprofloxacin	S≤0.03 R≥0.12	128(98.5)	0	2 (1.5)	0.004	0.008	0.001–0.12

S: susceptible, I: intermediate, R: resistant.

**Table 3:** Molecular characterization of ciprofloxacin-resistant *Neisseria meningitidis* isolates from Argentina.

Isolate	Year	City, Province	Characterization	CIP- MIC (mg/L)	Mechanism of CIP resistance	Reference
2026	2002	Cipolletti, Rio Negro	Y:NT:P1.5^a^	0,12	Efflux	[18]
2136	2003	La Plata, Buenos Aires	B:1:NST^a^	0,06	gyrA Asp-95 Asn	[18]
2417	2005	La Plata, Buenos Aires	B:NT:P1.13^a^	0,06	gyrA Asp-95 His	[45]
3032	2010	Bariloche, Rio Negro	W135∶2a:P1.5,2:ST-11	0,12	gyrA Thr-91-Ile	This study
3052	2010	Buenos Aires city.	W135∶2a:P1.5,2:ST-11	0,12	gyrA Thr-91-Ile	This study
3156	2011	Mendoza	W135∶2a:NST:ST-11	0,25	gyrA Thr-91-Ile	This study
3264	2011	Santa Fe	W135∶2a:P1.5,2:ST-11	0,12	gyrA Thr-91-Ile	This study
3324	2011	Pilar, Buenos Aires	W135:NT:P1.5,2:ST11	0,06	In study	This study

a Sequence type not determined, CIP: ciprofloxacin.

## Discussion

The increase of serogroup W135 in the last few years represented a major change in the epidemiology of meningococcal disease in Argentina. Seven isolates from 2008 presented the genotype W135∶2a:P1.5,2:F1-1:ST-11:ST-11 complex, which were indistinguishable from the strain associated with an international outbreak among Hajj pilgrims in 2000 and 2001 [19], [20], [21]. This strain was also the most prevalent among disease-associated isolates in Argentina in 2010, and was the cause of disease in 26% of isolates processed at the NRL. Despite the increase on the number of IMD cases caused by this strain, the overall number of cases and the number of isolates sent to the NRL for characterization have not changed substantially, suggesting that this strain has displaced other pathogenic strains. The combination W135∶2a:P1.5,2, which was found in 54% of our W135 isolates, appears to be also circulating among other countries in Latin America [22], [23], [24], [25] (Ana Belén Ibarz-Pavón, personal communication). During the course of the study, a problem was encountered with the MAb for the serosubtype P1.5 variant, as a large number of isolates showed low reactivity to this antibody. Therefore, the implementation of serosubtyping by *porA* sequencing techniques would be advisable in Latin American countries, especially since this protein is included in a number of Outer Membrane Protein (OMP) based vaccines currently under investigation. Analysis of additional vaccine-related OMPs, such as fHbp, nadA and nHba, will be considered in the near future once sequencing methodologies are well established in the laboratory and access to reagents and consumables needed for this methodology becomes steady in Argentina.

Serogroup B was the most prevalent serogroup in Argentina between 2001 and 2008, and was the second most prevalent among the 2010 isolate collection presented here. Most serogroup B isolates detected during 2010 belonged to the ST-3327:ST-865 complex, and this observation was consistent with the distribution found in a previous study among serogroup B disease-causing isolates from 2006, where 13 of the total 46 isolates for which MLST and *porA* sequencing data were obtained were characterized as B:21,16–36:ST-3327:ST-865 complex (unpublished data). Likewise, a study carried out among serogroup B meningococci isolated between 2001 and 2003 used *porA* sequencing as an alternative to ELISA, as many isolates were found to be NST with the currently available battery of MAbs. Serosubtype 21,16–36 was found in 30% of the isolates of that period [26]. As no MAb is available for the P1.21 variable region, and the P1.16–36 is not recognized by the P1.16 MAb, the only way to identify this serosubtype is by *porA* sequencing. It is likely that the NST ST-3327:ST-865 isolates from the 2010 collection presented that particular serosubtype, but *porA* sequencing would be needed for confirmation.

Serogroup B disease isolates from Europe and the United States frequently belong to the ST-41/44 complex, and to a lesser extent, ST-32 and ST-269 complexes [27], [28], [29], [30]. In contrast, only 4 (7%) of the serogroup B isolates obtained in Argentina in 2010 belonged to ST-41/44 complex, two of which, ST-9437 and ST-9439 were identified here for the first time. The ST-32 complex was poorly represented in the dataset, and no ST-269 complex isolates were detected. The ST-35 complex was found in 12 (21,5%) of serogroup B isolates, with most of them presenting the phenotype B:4:P1.22-1,14. This observation is also consistent with what was found among 2006 isolates, were ST-35 represented 21,6% of isolates and all of them were also genosubtyped as P1.22-1,14 (Unpublished data). The only ST-103 isolate found belonged to serogroup B and presented a different serotype:subtype combination to the C:23:P1.4–6 strain that has caused several recent outbreaks in Brazil and prompted the introduction of the serogroup C conjugate vaccine (MenC) in 2010 [31], [32]. There was a high proportion of NST isolates in both ST-35 and ST-865 complex. Given the high variability and constant evolution of OMP, implementation of genetic characterization of vaccine-related OMPs (fHbp, nadA, nHba and porA) is needed, especially in countries where diversity data is scarce, as upcoming meningococcal vaccines are being developed on these bases.

Only 7 serogroup C isolates where found among this strain collection. Three isolates presented the combination C:2a:P1.5:ST-11:ST-11 complex, which is undistinguishable from the strain that caused the increase of disease in the United Kingdom in the mid-1990s that resulted in the implementation of the MenC in the National Vaccination Schedule in 2000 [33]. More so, these three isolates might suggest a possible capsule switch between serogroup C and W135, as they are indistinguishable from some of the W135 isolates found among the 2010 isolate collection. Evidence of capsule switching is also seen in the W135:NT:NST:ST-3327, as this serotype:serosubtype combination appears to be more frequently associated to a serogroup B capsule among our isolates.

One NG isolate was found in an immunocompetent patient, belonging to the ST-198 complex, which is associated with the capsule null locus [34], [35]. Further examination of this isolate using molecular techniques is being done to determine whether this is capsule-null strain [36], [37], [38].

The high prevalence of the hypervirulent clone W135∶2a:P1.5,2:ST-11:ST-11 complex in Argentina indicates that any vaccination strategy needs to consider the inclusion of this serogroup in the vaccine formulation. Also, if bacterial transmission is to be stopped, it is important that vaccines are made available to all those included in high-risk groups, and not just those who can afford it in the private sector. With the upcoming licensure of protein-based universal vaccines, data is urgently needed for an evidence-based decision on their introduction in Argentina, as well as to identify at-risk populations and ensure a cost-effective vaccination strategy. As none of the major serogroup B genotypes associated with invasive disease in Europe and the United States appear to be relevant among the Argentinean strains, it is possible that vaccine compositions based on OMP diversity detected among currently available strain collections, which have little representation of Latin American strains, might not be suitable for their use in this continent. This hypothesis might be further supported by the fact that 10.4% of isolates belonged to previously unreported STs, as association between clonal complex and some of the OMPs, specifically, porA and fetA, has been described before [12]. It is important to highlight that serogroup B is still the second disease-causing serogroup Argentina, and it is also the first cause of disease in other Latin American countries [5] and, therefore, a prevention strategy against this serogroup should still be taken into consideration when available.

As expected for NM, all strains remain susceptible to ceftriaxone, chloramphenicol, tetracycline and rifampin. When susceptibility data is analysed reveals that 35.4% of the isolates showed intermediate resistance to penicillin, which could be attributed to the presence of mutations in the *pen*A gene, the main mechanism of penicillin no-susceptibility in NM [39]. The prevalence of intermediate penicillin resistance is low if we compare these data with that obtained in Argentina in 2006, where 51/65 (78.5%) of the NM isolates presented this phenotype of resistance (Alejandra Corso, personal communication). The decrease in this resistance found between 2006 and 2010 was clearly associated with the replacement of serogroup B by W135 observed during this period of time [7]. According to data from the National Surveillance Program 1998–2010, serogroup B in Argentina was associated with nearly 90% of intermediate penicillin resistance isolates, whereas only 10% of serogroup W135 isolates displayed this resistance.

The reduced susceptibility to ciprofloxacin in NM has been associated with point mutations in the QRDRs of the target sites for the fluorquinolones and specifically with changes in the GyrA subunit of DNA gyrase [40]. This paper describes twoW135∶2a:P1.5,2:ST-11:ST-11 complex isolates displaying resistance to ciprofloxacin associated with a mutation in the QRDR of *gyr*A gene Thr91-Ile. Previously, Corso *et. al.* reported the emergence of decreased susceptibility to ciprofloxacin in three clinical isolates from Argentina [18], [41]. In those cases, the resistance was associated with a mutation in the QRDR of *gyr*A gene (Asp95-Asn and Asp95-His) in two isolates (B:B1:P1.NT and B:NT:P1.13 respectively) and an efflux pump in the other one (Y:NT:P1.5) (Table 3). The majority of the NM with no susceptibility to ciprofloxacin reported to date present *gyr*A mutations, and the alteration at Thr91-Ile seems to be the most common [40], [42]. This mutation was originally described in Spain 2003 [43] and subsequently found in 11 serogroup A strains from New Delhi, India [44], [45], as well as in three serogroup B strains from the USA [42], [46], [47]. The non-susceptibility to ciprofloxacin was detected for first time in serogroup W135 during 2008 in two NM strains from Spain (Thr91-Ile) and in one from France (Thr91-Ile) [40], [48]. None of the 8 isolates studied in Argentina presented mutations in the *par*C subunit. Interestingly, three new cases of serogroup W135 NM isolates belonging to the ST-11 clonal complex and presenting resistance to ciprofloxacin were detected through the ArgentineanNational Surveillance Program during 2011. Two of them presented the same QRDR of *gyr*A Thr91-Ile mutation, while the remaining one is currently under investigation (Table 3).

In conclusion, data from meningococcal isolates obtained from disease cases in 2010 in Argentina confirm the presence of the epidemic clone W135∶2a:P1.5,2:ST-11 in the country and highlights the need to consider the inclusion of this serogroup in any vaccination strategy, notwithstanding the importance of a prevention strategy against serogroup B disease. Moreover, the presence of this strain in other countries in Latin America is to be expected, and given its epidemic nature, close monitoring and surveillance of meningococcal disease is recommended across the continent. The identification of new STs in this dataset evidences the necessity to implement molecular characterization of isolates in Argentina and Latin America. As new vaccine formulations are being developed solely on the basis of molecular data, it is imperative that molecular epidemiology data from Argentina, and indeed from Latin America, is made available for comparison to that of other regions in the world. The changes in the epidemiology of meningococcal disease occurred in Argentina in recent years, and the emergence of new mechanisms of antibiotic resistance, such as fluorquinolones resistance, highlight the necessity and importance of maintaining and strengthening National Surveillance Programs, and the need to implement new characterization techniques that allow data to be comparable to that reported from other regions in the world.
